# Effects of Dielectric Properties of Human Body on Communication Link Margins and Specific Absorption Rate of Implanted Antenna System

**DOI:** 10.3390/s25113498

**Published:** 2025-05-31

**Authors:** Soham Ghosh, Sunday C. Ekpo, Fanuel Elias, Stephen Alabi, Bhaskar Gupta

**Affiliations:** 1 Department of Electronics and Telecommunication Engineering, Jadavpur University, Kolkata 700032, India; bhaskar.gupta@jadavpuruniversity.in; 2 Communication and Space Systems Engineering Research Team, Manchester Metropolitan University, Manchester M1 5GD, UK; s.ekpo@mmu.ac.uk (S.C.E.); fanuel.elias@stu.mmu.ac.uk (F.E.); 3 SmOp Cleantech, Wilsons Park, Monsall Road, Manchester M40 8WN, UK; stephen.alabi@smopct.com

**Keywords:** biotelemetry, carrier link margin, data link margin, implantable antenna, meander line, specific absorption rate

## Abstract

**Highlights:**

This is the first work where the carrier link margin and the data link margin of a muscle-implanted antenna system with an external monitoring device are studied. The variation in the specific absorption rate (SAR) was investigated for a wide range of variations in the effective relative permittivity and conductivity of the human body.

**What are the main findings?**

- The carrier and data links margins of a biocompatible gelatin-encapsulated implantable medical device have been demonstrated.
- The performance matrices [including signal radiation (gain) and reflection (S_11_)] within the body model are comparable with the leading implantable antennas.
- The SAR value (220.26 W/kg) for a 1 g tissue at 1 W input power ensures the patient’s safety after implantation compared with the existing works.
- ANN modelling was performed to analyze the accuracy of the dependence. The innovative use of ANN in this field further underlines the pioneering nature of this research.

**What is the implication of the main finding?**

- The reported research findings will aid the innovative development of 5G/6G IoMT devices for existing and/or emerging wearable technology use cases and/or applications.

**Abstract:**

This study examines how the effective dielectric characteristics of the human torso affect the carrier-link-margin (CLM) and data-link-margin (DLM) of a biocompatible gelatin-encapsulated implantable medical device (IMD) that consists of a small implantable antenna, battery, printed circuit board (PCB), camera, and sensor operating at 2.5 GHz. The specific absorption rate (SAR) and the radio frequency (RF) link performances of the IMD are tested for ±20% changes in reference to the mean values of the effective relative permittivity, ɛ_eff_, and the effective conductivity, σ_eff_, of the human body model. An artificial neural network (ANN) with two inputs (ɛ_eff_, σ_eff_) and five outputs (SAR_1 g, SAR_10 g, fractional bandwidth, CLM, and DLM) is trained by 80% of the total scenarios and tested by 20% of them in order to provide reliable dependent analyses. The highest changes in 1 g SAR value, 10 g SAR value, fractional bandwidth, CLM, and DLM at a 4 m distance for 100 Kbps are 63%, 41.6%, 17.97%, 26.79%, and 5.89%, respectively, when compared to the reference effective electrical properties of the homogeneous human body model. This work is the first to accurately depend on the electrical analyses of the human body for the link margins of an implantable antenna system. Furthermore, the work’s uniqueness is distinguished by the application of the CLM and DLM principles in the sphere of IMD communication.

## 1. Introduction

**Figure 1:**
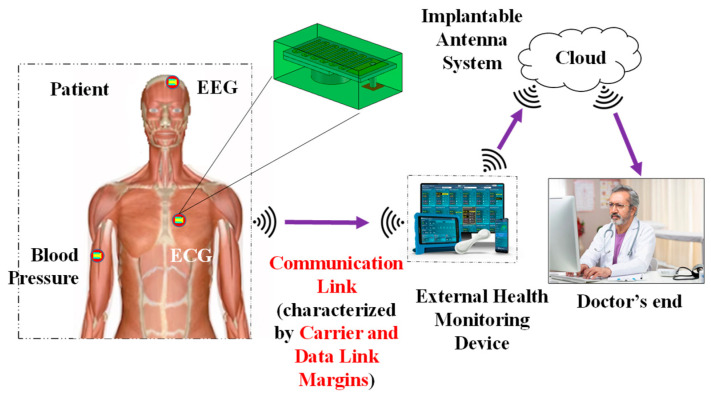
Overview of a wireless communication link between an implantable antenna system and an external health monitoring device.

The popularity of medical bio-telemetry devices over the past 20 years has increased interest in therapeutic and diagnostic features for ongoing patient monitoring in areas such as drug delivery, pain management, temperature, heartbeat, glucose level detection, and heart rate regulation [1,2,3]. With the aid of the new Internet of Medical Things (IoMT) technology, implantable antennas in these medical devices can wirelessly provide the doctor with health-related sensed data, potentially saving the patient from a critical condition or unexpected death [4]. Data loss due to inaccurate communication link characterization can result in a misdiagnosis and misinterpretation of the patient’s condition. The external monitoring device and implantable antenna gains, operating frequency, transmission power, path loss, antenna polarization, data rate, and signal-to-noise ratio (SNR) are all included in the quality of communication, also known as the link margin [5]. A wireless telemedical communication design between external monitoring equipment and an implanted antenna is depicted in Figure 1. In the past, implant communication was accomplished through inductive coupling. Limited data speeds (1–30 kbps), a short communication range (less than 10 cm), and limited coil sensitivity are some of the drawbacks of this communication method [6,7]. Medical Implant Communication Service (MICS) (402–405 MHz), Wireless Medical Telemetry Service (WMTS) (608–614, 1395–1400, and 1427–1432 MHz), and Industrial, Scientific, and Medical (ISM) bands (2.4–2.5 GHz, 5.73–5.88 GHz, etc.) are some of the frequencies used to get around these limitations [8,9].

Developing miniature prototypes, meeting safety regulations for specific absorption rate (SAR), and determining the dielectric characteristics of tissues are just a few of the major obstacles facing implantable antennas [10,11,12]. The SAR averaged over 1 g and 10 g of contiguous cubical tissue should be less than 1.6 W/kg and 2 W/kg, respectively, at 2.5 GHz, per electromagnetic regulatory recommendations [13,14,15,16,17]. Since the implantable antenna and monitoring device establish a wireless communication link via human tissue, the bioelectric characteristics of the tissue—namely, its relative permittivity and conductivity—determine the communication performance. Human tissue relative permittivity depends on a number of variables, including the patient’s age, gender, implant placement, skin and fat layer thickness, and hormone fluctuations [18,19,20,21,22]. It also depends on the fiber structure, water and oil content, etc. A portion of the skin layer’s epidermis, the stratum corneum, which is 0.4–0.6 mm thick, has relatively lower water content (around 10%) [23]. Hwang et al. [24] and Boric-Lubecke et al. [25] demonstrated that the effective complex permittivity of various bodily components varies. Effective complex permittivity may increase by 20% as a result of tumor growth in any layer. Pathological alterations in the liver, such as cirrhosis, steatosis, and hepatocellular cancer, can also alter the effective dielectric characteristics [26]. Therefore, taking into account differences of ±20% in the mean bioelectric characteristics of the entire human body is sufficient to investigate the potential variance in link performance.

Variations in the electrical characteristics of the implanted antenna’s biological surroundings can alter effective loss in channels, which could result in different communication connection performance metrics. The achieved gain and radiated power of the implanted antenna are modest for anatomical models with small sizes and low masses. In [27], it was evaluated how the reflection coefficient (S_11_) and fractional bandwidth of an implanted antenna at MICS (402–405 GHz) were affected by anatomical differences in the relative permittivity and conductivity of the human body model caused by variances in skin and fat thickness. The authors of [28] investigated how the relative conductivity of several tissues, including fat, kidney, liver, muscle, gray matter, and white matter, as well as the patients’ ages, genders, heights, and masses, affected the S_11_ and bandwidth of an implantable rectangular patch antenna. According to the literature review, the performance of implantable antennas is also influenced by the dielectric characteristics of human tissue. In [29], it was investigated how the relative permittivity and conductivity of the human body could detune an implanted antenna in terms of reflection coefficient. The authors of [3] examined how changes in the dielectric constant of the human head and body model affected the implanted antenna’s S_11_, resonant frequency, gain, and SAR. They only collected a very small number of samples for their experiments, which was insufficient to examine their dependencies. The data rate fluctuation, channel property uncertainty, etc., all affect the communication link.

An equivalent homogeneous human body model (mean tissue permittivity = 52.7 and mean conductivity = 1.95 S/m) was used to design a corner-chamfered meander-line implantable antenna in this article. The antenna’s communication performance was tested at the 2.45 GHz ISM band, which the FCC has designated for medical research. For every 20% difference in the mean relative permittivity and conductivity values of the human body, a 25 MHz shift in frequency from the reference frequency was noted. This study has examined how the positioning of implantable antennas may alter peak SAR and its distribution as a result of potential variations in bioelectrical characteristics. Input power, one of the primary characteristics for the link margin that characterizes the communication quality, must be appropriately adjusted in order to restrict the SAR value in accordance with safety regulations. Because it may describe the channel capacity of wireless communication using Shannon’s channel capacity theorem, the bandwidth of an implanted antenna is another crucial communication metric. The gain of the implanted antenna, the distance between the implanted antenna and the monitoring device, the gain of the monitoring antenna, and many communication variables such as the data rate, modulation type, and signal-to-noise ratio are all included in the link margin. The communication link margin is composed of two components: (a) the carrier link margin (CLM), which is associated with the carrier signal-to-noise ratio, and (b) the data link margin (DLM), which is associated with the data signal-to-noise ratio [29,30]. There is no published research on how the CLM and DLM depend on the SAR profile to provide safe and dependable wireless patient monitoring based on the efficient electrical characteristics of the body. By using an artificial neural network (ANN) to train and test the effective qualities with regard to the CLM and DLM, this paper fills a research need. A total of 80% of 2500 situations (±20% variations in the referenced mean relative permittivity, ɛ_eff_, and conductivity, σ_eff_, of the human body) were used to train the ANN, while 20% of them were used for validation. The average accuracy of the ANN’s predictions for both the simulated and measured responses was 99.24%. The largest differences in 1 g and 10 g SAR value, fractional bandwidth, CLM, and DLM at a 4 m distance for 100 Kbps are 63%, 41.6%, 17.97%, 26.79%, and 5.89%, respectively, when compared to the effective electrical properties of the homogeneous human body model.

An on-body and off-body communication system between wearable and implanted antennas at the ISM band (5.725–5.875 GHz) and the 5G n79 frequency band (4.4–5 GHz) is described by Sharma et al. in [31]. The ultra-wide impedance bandwidth of the planned antenna is 2.01 GHz. This article offers useful information about link analysis. Nevertheless, this work does not address the concepts of CLM and DLM. As indicated in Table 1, the suggested antenna system has a better communication link profile (in terms of communication range) than previously published studies [32,33,34,35,36]. There are no related works from the reported papers that investigate CLM and DLM.

Table 1 highlights the novelty of this work in comparison to earlier research published in [32,33,34,35,36,37,38,39]. Below is a summary of this work’s importance and timeliness.

(i) According to Table 1, this is the first study to examine the carrier link margin and data link margin of an external monitoring device on a muscle-implanted antenna.
(ii) In comparison to previous research, the SAR value (220.26 W/kg) for a 1 g tissue at 1 W input power guarantees the patient’s safety following implantation.
(iii) The body model’s performance matrices, which include signal radiation (gain) and reflection (S_11_), are on par with the top implantable antennas.
(iv) An implanted antenna with a communication range of up to 13 m was created by the authors in [36]. The impact of phantom size on the spiral-implanted antenna’s efficiency and gain was examined in [39]. Based on just six samples of permittivity values, it was possible that fluctuations in the relative permittivity of the body may cause variations in the S_11_ and frequency of an implanted antenna [36]. The impact of both human body electrical characteristics (such as effective relative permittivity and conductivity) on the link margins (CLM and DLM) is examined and validated in our work using a thorough methodology. The prevailing performance matrices for potential IoMT applications include operating frequency, realized gain, data rate, channel loss, the range of communication, bandwidth, and the SAR signature of the implanted antenna.
(v) To examine the correctness of the dependence, ANN modeling was conducted on 2500 samples of effective body models. The creative application of ANN in this area emphasizes the groundbreaking character of this study even more.
(vi) This study offers the first comprehensive examination of the electrical characteristics of the human body that depends on the SAR profile and implantable antenna communication link performance.
(vii) The presented study results will support the creation of 5G/6G IoMT devices for current and/or future wearable technology applications and use cases.

## 2. Models and Methods

### 2.1. Implantable Transmitting Antenna System Design

#### 2.1.1. Design Procedure

**Figure 2:**
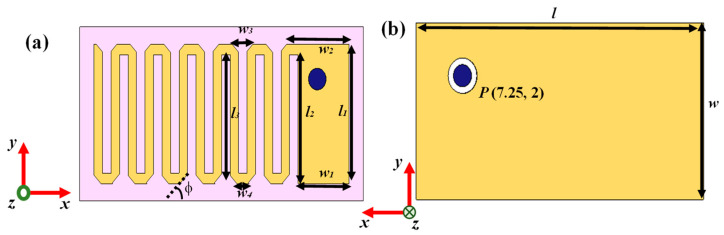
Geometry of the corner-chamfered implantable antenna: (**a**) top view and (**b**) bottom view.

**Figure 3:**
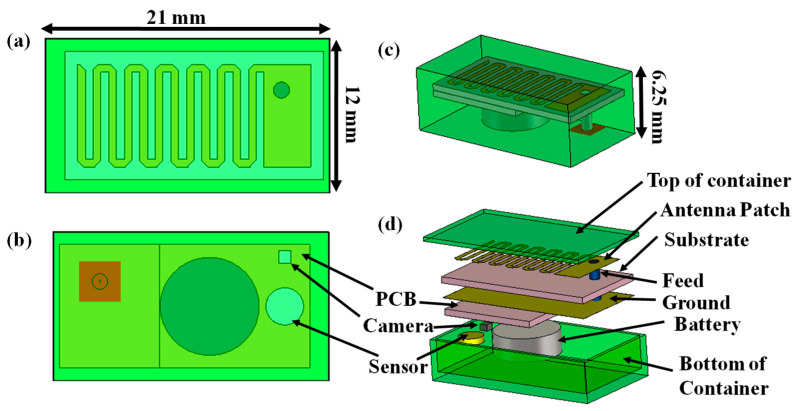
Detailed architecture of implantable device: (**a**) top view, (**b**) bottom view, (**c**) isometric view, and (**d**) exploded view.

The geometry of the designed 152.4 mm^3^ antenna within an implantable medical device is illustrated in Figure 2 and the parameters of the antenna are listed in Table 2. The radiator part of this antenna was printed on Arlon AD 430 (Arlon Electronic Materials, Delhi, India) dielectric substrates with a thickness of 0.762 mm. The meandering design was carried out here to lengthen the effective current path, which led to an increase in the effective wavelength with a miniaturized structure. Corner-chamfering was performed to reduce the stray radiated emission to minimize losses and unwanted reactance [40]. For a proper analysis of the communication and SAR performance, a prototype of a 21 × 12 × 6.25 mm^3^ implantable medical device (shown in Figure 3) containing the corner-chamfered antenna, camera, PCB, and sensor encapsulated by a 0.5 mm thick biocompatible coating made of 10 g gelatin (Kshipra Biotech Private Limited, Indore, India) and 15 g deionized (DI) water (Jadavpur University IC center, Kolkata, India) which has relative permittivity of 41.57 and loss tangent of 0.39 at 2.45 GHz, was obtained. A coaxial probe with a 0.63 mm probe radius providing an input impedance of 50 Ω was used for exciting the antenna system.

#### 2.1.2. Simulation Setup

**Figure 4:**
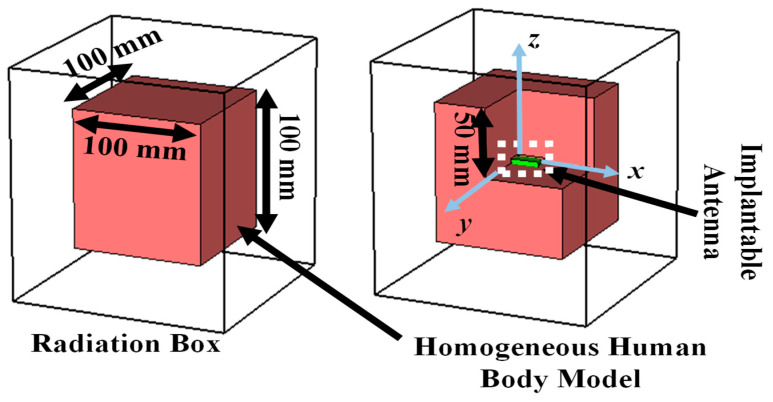
Homogeneous human body model considered to simulate implantable antenna system.

The corner-chamfered antenna was implanted within a 100 mm × 100 mm × 100 mm cubical mean homogeneous human body model (HHBM) at the center as shown in Figure 4. The HHBM has a relative permittivity of 52.7 and conductivity of 1.95 S/m based on the FCC guideline [41]. Generally, the human body is multilayered in nature and has different tissue layers such as skin, fat, blood, muscle, etc. The relative permittivities and conductivities of these layers at 2.5 GHz are mentioned in Table 3 as per Gabriel’s database [42]. It is very challenging to construct a multilayered phantom due to complexity, cost, and the possible formation of unwarranted air gaps between two layers or mixing chemicals of two layers while one layer is placed on another layer in a hot semi-solid form. However, this problem can be resolved by using its equivalent homogeneous phantom. There is no possibility of generating air gaps inside a homogeneous phantom; the cost and complexity of designing a homogeneous phantom are comparatively lower than a multilayered model. The HHBM was enclosed by an airbox, which is considered as the radiation boundary of the implanted system. Numerical simulations were performed using the finite element method (FEM) within the CST MWS 2019 commercial software(Licensed Version). Here, a Gaussian source was fed to the implanted antenna and the frequency response was measured at 2.5 GHz.

#### 2.1.3. Design Evolution

**Figure 5:**
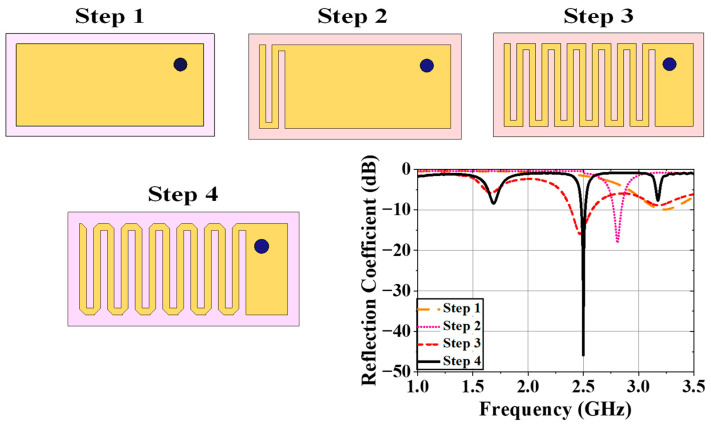
Steps in the design of the implantable antenna within the human body model and their reflection coefficients (dB).

The design steps of the implantable antenna are shown in Figure 5. An initial patch with a size of 18 mm × 8 mm was designed on a substrate, resulting in a poor S_11_ response at an operating frequency of 3.18 GHz. In Step 2, a meander line was created at the opposite edge of the excited part by etching two 0.6 mm slots as shown in Figure 5. The operating frequency was shifted to 2.82 GHz and S_11_ was improved to −18.05 dB. The incorporation of slots increased the effective guided wavelength by increasing equivalent capacitance. To tune the operating frequency within the 2.45 GHz ISM band (2.4–2.5 GHz), more symmetrical slots were etched to increase meander-line numbers. After creating six meander-line slots as shown in Figure 5, the operating frequency was tuned at the desired band. The reflection coefficient (S_11_) of this antenna architecture was observed as −15.59 dB. The realized gain of this antenna was −47.28 dBi. To improve the antenna responses in terms of S_11_ and gain, the chamfering of the corners was performed. The operating frequency was not varied due to chamfering; however, the reflection coefficient was improved to −45.87 dB and the realized gain was increased to −38.42 dBi. The simulated antenna possessed a fractional bandwidth of 5.67%.

#### 2.1.4. Current Distribution and SAR Profile

**Figure 6:**
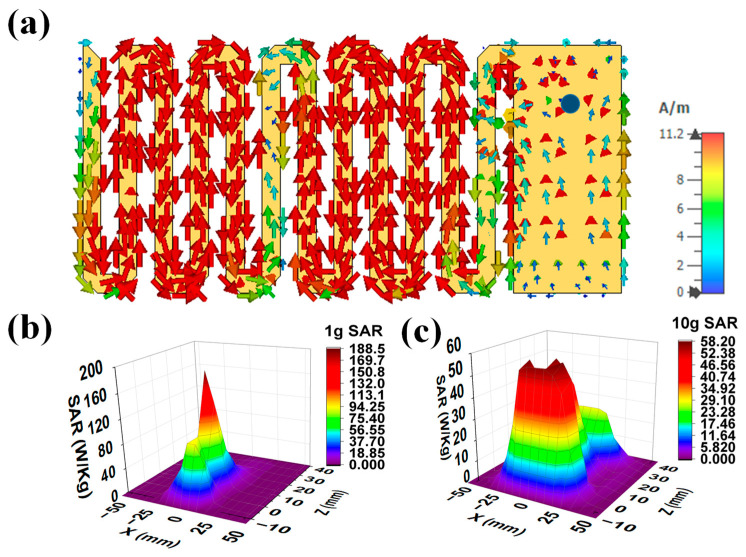
(**a**) Surface current distribution on a patch of antenna at 2.5 GHz; 3D 1 W SAR plots (Y = 0 calibration) for (**b**) 1 g and (**c**) 10 g tissue.

Figure 6a shows the surface current distribution of the final antenna radiator at 2.5 GHz. This current was injected through a 50Ω coaxial probe from an RF source. From this figure, it can be seen that the current was distributed from the connector to the patch surface. The currents were highly dense at the edges of meanderlines. The safety performance of the implanted antenna was evaluated in terms of compliance with international safety guidelines, the IEEE Std. C95.1-2019 [41] guideline. Initially, a 1 W average input power was applied to the implanted antenna to observe the SAR distributions for 1 g and 10 g contiguous models. The maximum SAR for the 1 g and the 10 g models should be less than 1.6 W/kg and 2 W/kg, respectively, as per the standard guidelines [41]. The peak SAR values for the 1 g and 10 g cubical tissues were obtained as 220.26 W/kg and 59.73 W/kg, respectively. The maximum input powers for 1 g and 10 g of body tissues were 7 mW and 24 mW, respectively, for maintaining the SAR values within the standard SAR limits. Figure 6b,c present a simulated 3D 1 g and the 10 g SAR distribution for the implanted antenna, respectively, for a 1 W input power.

### 2.2. Antenna Design for Monitoring Device

**Figure 7:**
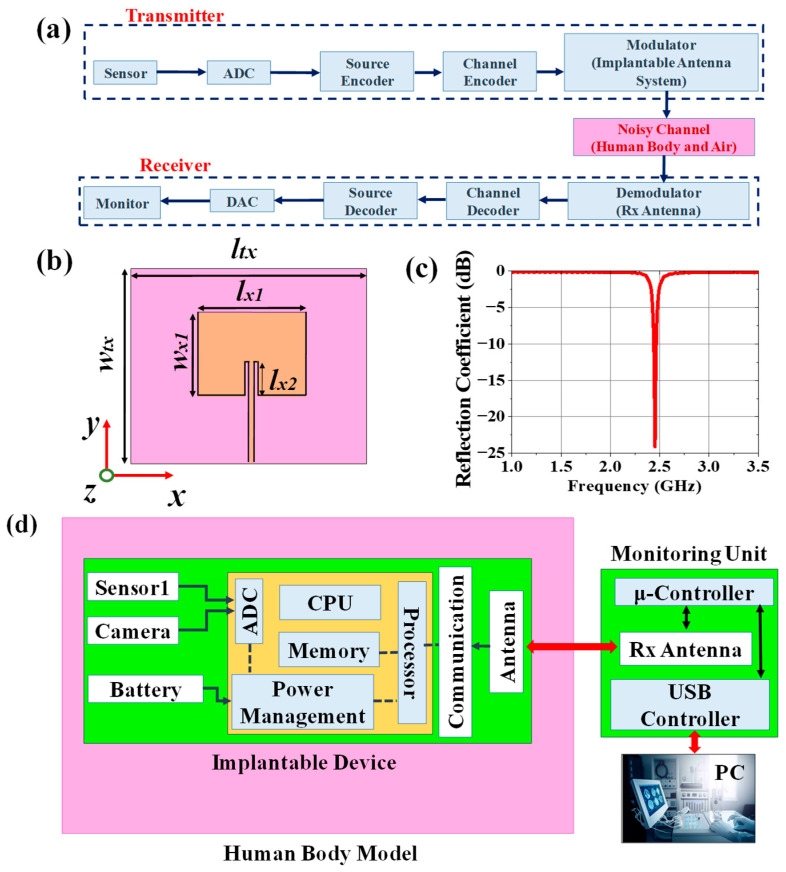
(**a**) General block diagram of wireless patient monitoring. (**b**) Top view of receiving antenna present in external monitoring device where *l_tx_* = 90 mm, *w_tx_* = 80 mm, *l_x_*_1_ = 38 mm, *w_x_*_1_ = 29 mm, and *l_x_*_2_ = 7.5 mm. (**c**) Reflection coefficient plot of Rx antenna. (**d**) Hardware schematic of the system.

**Figure 8:**
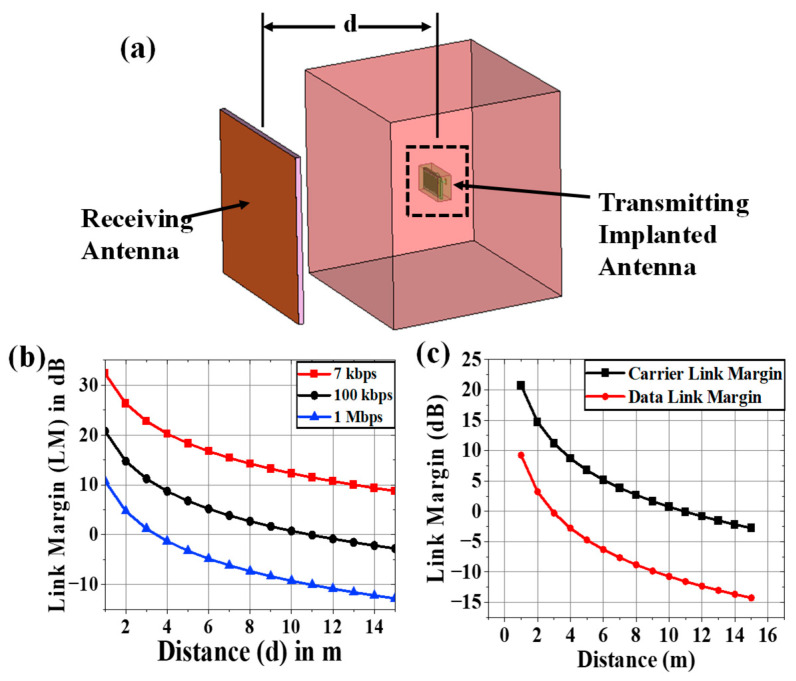
(**a**) Simulation setup for calculating link margin, (**b**) generalized link margin vs. distance between implanted antenna system and receiving antenna, and (**c**) CLM and DLM vs. distance.

The external monitoring device can wirelessly monitor the patient’s health condition by collecting sensed data from the implantable antenna system tuned to the antenna of the external receiving device as illustrated in Figure 7a. Therefore, the receiving antenna of the external monitoring device should be tuned at 2.5 GHz. A patch antenna was designed and placed at a 50 mm distance from the top surface of the phantom (refer to Figure 8a). The top view of the receiving patch antenna is shown in Figure 7b. The antenna has a return loss of 24.09 dB at an operating frequency of 2.49 GHz, with a realized gain of 4.95 dBi.

### 2.3. Communication Performance Characterization

For the proper establishment of medical communication with external monitoring devices to conduct continuous patient monitoring, the link margin (L) should be calculated using (1). The hardware schematic of the system is shown in Figure 7d. Based on the SAR profile, the input power of the implantable antenna will be P_TX_ = 7 mW = 8.45 dBm.

L = R_Link_ (dB) − R_req._ (dB) (1)

R_Link_ = P_TX_ + G_TX_ + G_RX_ – 20 × log(4πd/λ) (2)

R_Req._ = (E_b_/N_o_) + 10 × log(D_r_) − G_C_ +G_D_ (3)

In (2), the expression R_Link_ represents the ratio of the Rx power the patch antenna receives at any distance (d) to the noise power density of the implanted chamfered meander-lined antenna. R_Req_, in (3), denotes the ratio of required carrier power to noise power at the Rx end [43,44]. Binary phase shift keying (BPSK) modulation with a normalized signal-to-noise ratio (E_b_/N_o_) of 9.6 dB was considered here, and L was calculated using the link budget parameters mentioned in Table 4 [43] for bit rates of 7 kbps, 100 kbps, and 1 Mbps. From Figure 8b, it was observed that the implantable antenna is able to communicate with the Rx antenna at a distance of more than 15 m for a bit rate of 7 kbps. If the value of Dr is increased to 100 kbps, the transfer range (where L (dB) = 0) is reduced to 10 m. For a data rate at 1 Mbps, an implantable antenna communicates efficiently within 3.5 m.

### 2.4. Carrier Link Margin and Data Link Margin Calculation

Noise temperature is one of the important parameters of any system. Noise temperature is the measurement of the equivalent level of available noise power introduced by a component or source. Let us consider the effective noise temperature of the system, which is T_sys_ [45]. Therefore, the modified expression of the generalized link margin is given in (4) [43,44,45].

L = P_TX_ + G_TX_ + G_RX_ − 20 × log(4πd/λ) − (E_b_/N_o_) − 10 × log(D_r_)+G_C_ − G_D_ − 10 × log (Tsys) (4)

To characterize the wireless communication link between the implantable antenna and an external device, the carrier link margin (CLM) and data link margin (DLM) were required to learn. The CLM is the difference between the achieved carrier-to-noise power and the required carrier-to-noise power in human body communication, and the DLM is the difference between the data signal-to-noise power achieved and the data signal-to-noise power required. The CLM and DLM are calculated using (4) for a 14 K noise temperature and 100 Kbps data rate and plotted in Figure 8c for variable distance (d) between the receiver and transmitting implanted antenna.

## 3. Dependence Analysis and Discussion

The human body model’s respective mean relative permittivity (ɛ_eff_) and conductivity (σ_eff_) are 52.7 and 1.95 S/m as per FCC guidelines at 2.5 GHz. The electrical properties can be varied by around ±20% with respect to reference ɛ_eff_ and σ_eff_. Therefore, two Gaussian pulses (N_ɛ_ and N_σ_) for ɛ_eff_ and σ_eff_ were taken as mentioned in Equations (5) and (6) and sampled into 50 points. Therefore, 2500 scenarios with different combinations of ɛ_eff_ and σ_eff_ were obtained to record the implanted antenna’s communication performance and SAR profile. The possible variations in medical communication performance and SAR profile due to both body-electrical properties variations were studied here.

N_ɛ_ = {1/√(2π(Ψ_ɛ_)^2^} × exp[(−1/2){(ɛ_eff_ − 52.7)^2^/(Ψ_ɛ_)^2^}] (5)

N_σ_ = {1/√(2π(Ψ_σ_)^2^} × exp[(−1/2){(σ_eff_ − 52.7)^2^/(Ψ_σ_)^2^}] (6)

### 3.1. Variation in Effective Relative Permittivity

**Figure 9:**
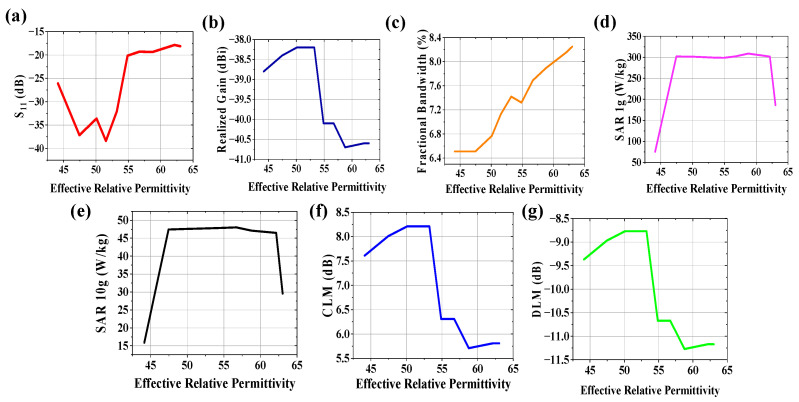
Effect of +20% to −20% variation in effective relative permittivity on (**a**) S_11_, (**b**) realized gain, (**c**) fractional bandwidth, (**d**) 1 g SAR (1watt input power), (**e**) 10 g SAR (1 watt input power), (**f**) CLM (100 kbps data rate at d = 4 m), and (**g**) DLM (100 kbps data rate at d = 4 m) of the implanted antenna system.

The communication channel characteristics between an implantable antenna system and external receiving antenna connected to a monitoring device are varied due to variation in electrical property of human tissues. The effect of the effective relative permittivity of the body model on radiation performance (S_11_, realized gain, and fractional bandwidth), communication performance (CLM and DLM for a 100 kbps data rate at 4 m from the implantable antenna system), and SAR profile (SAR for 1 g and 10 g contiguous tissue) was studied at 2.5 GHz, keeping the effective conductivity of the body model constant at 1.95 S/m, and plotted in Figure 9. Due to variation in the effective relative permittivity of the human body, the return loss is improved for 44.12 < ɛ_eff_ < 55 due to an increase in loading on the antenna system. Beyond this range, the impedance loading does not have adominant effect on the reflection coefficient of the system, as obtained from Figure 9a. For 44.12 < ɛ_eff_ < 55, variation in the realized gain (Figure 9b) is almost negligible (~38.4 dBi). However, it is deteriorated towards −41 dBi, which also leads to worse CLM and DLM (refer to Figure 9f,g). The misinterpretation of a patient’s data, noisy data acceptance, may happen at the receiving end. Therefore, a doctor cannot properly monitor and diagnose a patient wirelessly. From Figure 9c, fractional bandwidth is improved with an increase in effective relative permittivity. SAR values for both 1 g and 10 g tissue (Figure 9d,e), respectively) are not widely dependent on effective relative permittivity variation between 47.45 < ɛ_eff_ < 62.15.

### 3.2. Variation in Effective Conductivity

**Figure 10:**
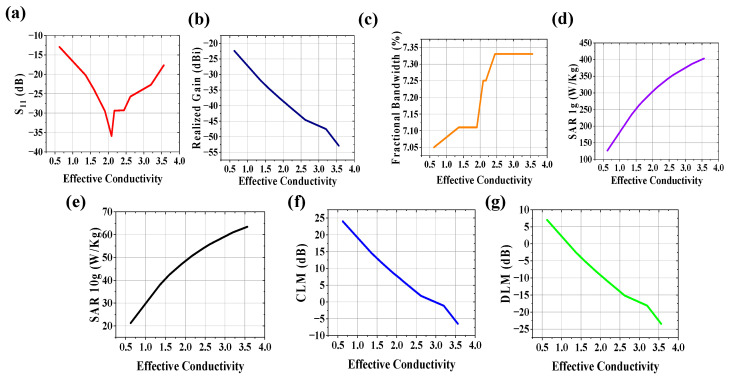
Effect of +20% to −20% variation in effective conductivity on (**a**) S_11_, (**b**) realized gain, (**c**) fractional bandwidth, (**d**) 1 g SAR (1watt input power) (**e**) 10 g SAR (1 watt input power), (**f**) CLM (100 kbps data rate at d = 4 m), and (**g**) DLM (100 kbps data rate at d = 4 m) of the implanted antenna system.

The effect of the effective conductivity of the body model on system performance was studied at 2.5 GHz, keeping the effective relative permittivity of the body model constant at 52.7, and plotted in Figure 10. The increase in the conductivity of the wireless communication channel can increase noise in terms of loss tangent (tan δ) = σe_ff_/ωε_eff_. From Figure 10a, it is seen that the S_11_ value is improved when σ_eff_ is between 1.5 and 3 S/m. The realized gain (Figure 10b), CLM (Figure 10f) and DLM (Figure 10g) deteriorates with an increase in the conductivity of the human body, which hampers the communication performance for the wireless body area network. Analyzing the plot of fractional bandwidth vs. effective conductivity shown in Figure 10c, it is observed that fractional bandwidth has an almost linear relationship with effective conductivity with low slope. SAR values for both 1 g and 10 g tissue (Figure 10d,e, respectively) are highly dependent on effective conductivity variation. SAR values increase on a large scale with conductivity increases, which may hamper patient safety.

### 3.3. Variations in Both Effective Relative Permittivity and Conductivity

Communication characteristics, such as CLM and DLM, include the realized gain and reflection coefficient of the implantable antenna system. Therefore only CLM, DLM, SAR for 1 g and 10 g tissue models, and fractional bandwidth are considered for dependence analyses. A total of 2500 scenarios (±20% variations in reference to the effective relative permittivity and conductivity of the human body) were considered here. In Ghosh et al. [46], it was discussed that the polynomial fitting technique and statistical analysis could not estimate the implantable antenna performance, especially fractional bandwidth, when more than one variable was considered. This is due to the highly uncertain nature of human body tissues. An ANN can estimate these performance parameters very efficiently. That is why an ANN-based model was considered here over traditional methods when both effective relative permittivity and conductivity were considered as variables.

**Figure 11:**
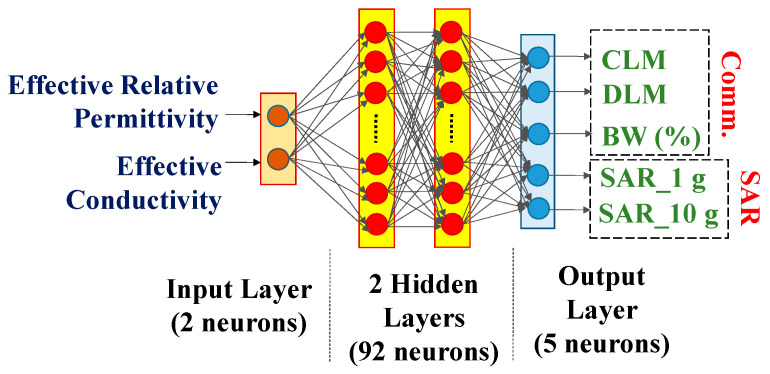
Artificial neural network.

An artificial neural network (ANN) with two input neurons (ɛ_eff_ and σ_eff_), two hidden layers (92 neurons), and five output layers (CLM, DLM, BW (%), SAR_1 g, and SAR_10 g), shown in Figure 11, was trained with 80% of the total scenarios and validated by the remaining 20% of the data. In the output layer, the activation function was seen as a “piecewise-linear function”; however in the other layers, it was regarded as a “rectified linear unit (ReLU)”function. The mean absolute error (MAE) [46] was selected as the loss function in this study. The learning rate at the start was 0.01. After 300 epochs of recording, the training converged to 0.46. On an AMD Ryzen 5 5500U (AMD Industries Limited, Delhi, India) with Radeon graphics running at 2.10 GHz and 8 GB of RAM, the training took about one and a half minutes [46]. It took two milliseconds every step to train. In CST, ten arbitrary scenarios were simulated, and simulated answers were gathered. To observe the effectiveness of the developed ANN’s prediction, the reactions of identical circumstances that the ANN anticipated were recorded. The ANN was modeled to predict the antenna system performance parameter values for any random set of (ɛ_eff_, σ_eff_) because the set values are different for different patient bodies. With the variation in the effective relative permittivity of the human body model, the impedance performance was affected and electric field distributions within human tissue were varied. Therefore, the SAR was greatly influenced with the variation in effective relative permittivity. The CLM and DLM were not highly influenced with the variation in effective relative permittivity because permittivity variation did not directly affect the gain in the implantable antenna system. With the variation in effective conductivity, the SAR values were highly influenced because the SAR was proportional to the conductivity of the environment of the system. Due to this variation, the variations in CLM and DLM values were also noticeable because the effective loss of the communication channel between implanted antenna system and external monitoring device increased with the increase in the conductivity of the human body, which reduced the gain and link performances (CLM and DLM) of the implanted system.

## 4. Experimental Setup and Measurement

### 4.1. Implantable Antenna System

**Figure 12:**
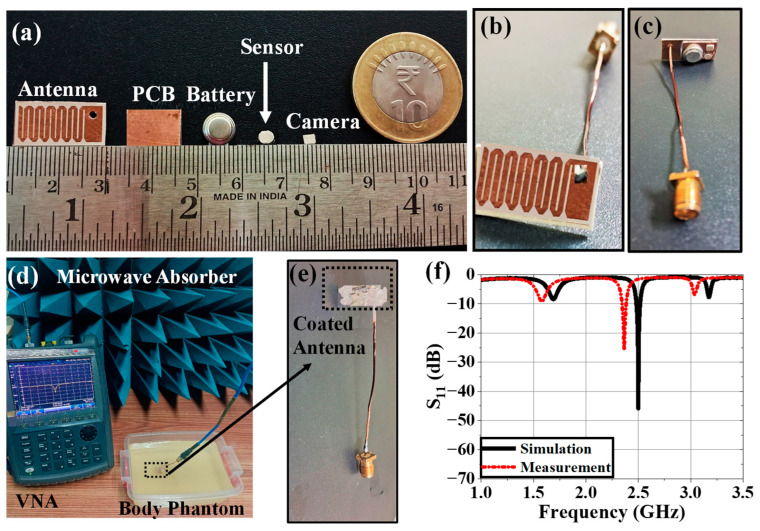
(**a**) Fabricated prototypes of all components of the implantable antenna system, (**b**) top and (**c**) bottom views of implantable antenna system without cover, (**d**) reflection coefficient measurement setup of implantable antenna, (**e**) top view of antenna system within gelatin coating, and (**f**) comparison between simulation and measurement.

In this section, the in vitro performance analysis of the simulated implantable antenna system is described. The physical prototypes of implantable antenna and receiving antenna were fabricated. The fabricated prototype of the implantable antenna is shown in Figure 12a. A small battery (π × 3.952 × 3.6 mm^3^), prototype of PCB (Arlon AD 430 covered with copper in both sides), and prototypes of the sensor and camera (Arlon AD 430) were used to replicate the simulated antenna system as shown in Figure 12a. To excite the antenna, a 50 Ω coaxial cable was connected using soldering (Figure 12b,c) and the other end was connected with one of the two ports of the vector network analyzer (VNA) for S-parameter measurement as illustrated in Figure 12d. Top and bottom views of the implantable antenna system before coating are shown in Figure 12b and Figure 12c, respectively.

**Figure 13:**
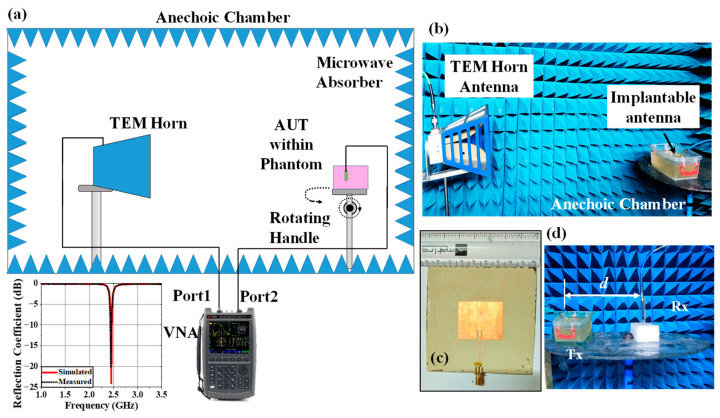
(**a**) Schematic of far-field radiation pattern measurement setup within anechoic chamber and its required hardware. (**b**) Far-field radiation pattern measurement of implantable antenna system within phantom using a TEM horn antenna. (**c**) Top view of fabricated receiving antenna and its reflection coefficient plots (comparison between simulation and measurement). (**d**) Communication performance between Tx implantable antenna system and Rx antenna.

**Figure 14:**
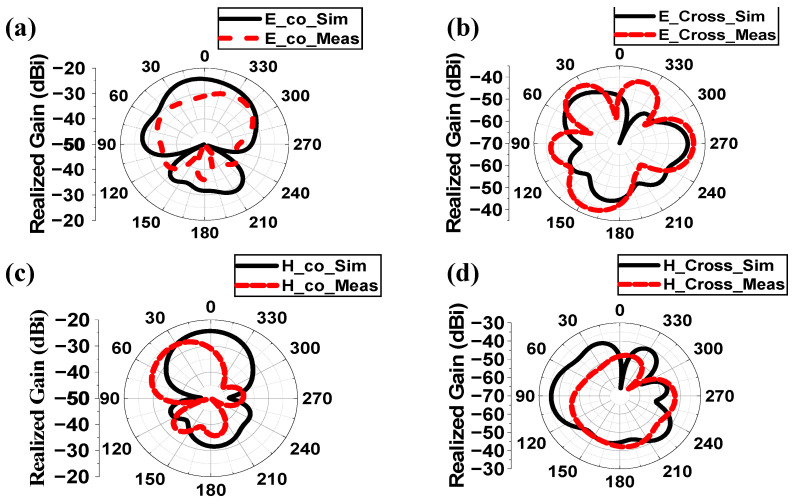
Comparison of radiation patterns between simulated and measured responses of implantable antenna system within human phantom at (**a**) E-plane co-polar, (**b**) E-plane cross polar, (**c**) H-plane co-polar and (**d**) H-plane cross polar at 2.5 GHz.

The whole system was covered with a biocompatible gelatin cover made of 15 g DI water, and 10 g gelatin, which is depicted in Figure 12e. For in-vitro measurement, a homogeneous semi-solid body phantom model was developed by 66.3% DI water, 31.6% DGME, 0.4% NaCl, and 1.7% agar agar powder. The dielectric properties in terms of the relative permittivity and conductivity of the phantom were measured using an Agilent 85070E dielectric kit. The developed phantom had an effective relative permittivity and conductivity of 51.75 and 1.91 S/m, respectively, at 2.5 GHz. The designed antenna system was then implanted within a semi-solid human body phantom at a 50 mm depth from the top surface, as shown in Figure 12d, to measure the reflection coefficient of the implanted antenna. The comparison between simulated and measured S_11_ responses was plotted in Figure 12f. The measured S_11_ was −22.97 dB at 2.43 GHz. The co- and cross-polar E and H plane far-field radiation patterns obtained from the measurement setup shown in Figure 13a,b are plotted in Figure 14a–d. The antenna system had a measured realized gain of −32.68 dBi at its operating frequency.

In this work, the size of the implantable antenna system (21 × 12 × 6.25 mm^3^) was very small with respect to the phantom container size (100 × 100 × 100 mm^3^) where antenna system was placed at a depth of 50 mm from the bottom of the container. The antenna’s far-field distance did not include the container walls. Consequently, the impedance performance of the implanted antenna system could not be affected by the thicknesses and materials of the container. Because agar agar powder was added to the phantom recipe, the phantom was not liquid. To lessen the chance of implantable antenna systems misaligning while detecting radiation patterns, the semi-solid phantom was created. To determine the radiation pattern of the implanted antenna system, the container was setup on a turntable, which spins at various angles between 0° and 360°. The phantom’s relative permittivity is 52.7, which is extremely high when compared to plastic material, and the container’s relative permittivity is nearly 2.2. In terms of the phantom, the plastic container’s conductivity is essentially nonexistent. When measuring the radiation pattern, the phantom’s thickness in the broadside direction of the antenna system was 50 mm, and the plastic container’s thickness was approximately 1 mm. As a result, the container’s impact on the system’s radiation pattern inside the phantom was minimal.

For both the E and H planes in the simulation, the implanted antenna’s major lobes in the human body phantom in Figure 14 were oriented broadside. However, the primary lobes were somewhat skewed from their simulated directions because of a little misalignment during the measurement period and the existence of various losses, including cable and connector losses, among others. Additionally, there were some side lobes in both the simulation and measurement, suggesting that this antenna might also transmit in the directions of its end-fire.

Due to the lossy nature of the human body, the gain of an implanted system is diminished after it has been implanted. A very long communication range is not necessary for wireless patient monitoring. As a result, in real-world applications, the implantable antenna system’s low gain poses no issues. The surrounding tissue may become more heated as a result of the implantable antenna system’s high gain. It was evident from the connection margin study that the implanted antenna system could communicate up to 15 m, 10 m, and 3.5 m for 7 kbps, 100 kbps, and 1 Mbps data rate values, respectively. This was determined to be sufficient for appropriate wireless patient monitoring.

### 4.2. Monitoring Antenna

Similar procedures for measuring the radiation pattern and reflection coefficient as those used for implanted antenna systems were used to manufacture and measure the receiving antenna for the monitoring device that was designed in Section 2.2. Figure 13c displays the top view of the constructed Rx antenna as well as comparative graphs of the reflection coefficient for the measured and simulated findings. The manufactured antenna had an S_11_ and a broadside gain of 4.02 dBi and −20.18 dB. Since we are testing the gain of implanted antennas at various degrees while maintaining the Rx antenna in its fixed position (broadside direction), the entire radiation pattern of the Rx patch antenna was not measured. As seen in Figure 13d, the Rx antenna was placed at varying distances (d) from the implanted antenna in order to examine the communication performance. CLM, DLM, and gain were measured using the transmission coefficient.

### 4.3. Variation Analysis

By altering the quantity of DGME with 273 mL DI water and 794 mg NaCl, these situations were reproduced in a lab. By inserting a constructed antenna into ten prepared phantoms, the practical antenna parameters were gathered. The effective ANN predictions for the SAR profile and communication performance with changes in the human body model are displayed in Table 5 and Table 6, respectively. Table 5 and Table 6 show that, for ±20% potential differences in both electrical properties, the designed antenna could predict the implanted antenna’s communication performance with an average accuracy of 99.89% and 99.78%, respectively, based on simulation and testing. For 1 g and 10 g SAR profiles, the implanted antenna’s average accuracies were 99.01% and 98.27%, respectively. The highest changes in the 1 g and 10 g SAR values, fractional bandwidth, CLM, and DLM at a 4 m distance for 100 Kbps are 63%, 41.6%, 17.97%, 26.79%, and 5.89%, respectively, when compared to the reference effective electrical properties of the homogeneous human body model.

Due to ±20% potential fluctuations in dielectric characteristics, SAR values were found to vary greatly, potentially increasing patient safety concerns. In order to ensure patient safety, this work advises future engineers to take these ranges of variability into account while designing IMDs. Understanding CLM and DLM changes is also necessary to determine the extent to which noise or data degradation can occur, increasing the likelihood of inaccurate wireless patient monitoring.

To optimize the performance characteristics of IMDs for monitoring physiological data (blood flow, pressure, etc.), scientists should concentrate on the adaptive regulation of the system parameters in varied body environments in the future. Future studies assessing the efficacy and safety of IMDs in a real-world clinical setting may be another significant area of focus.

## 5. Conclusions

This study involved a numerical analysis of the effects of effective relative permittivity and conductivity on the communication performance and SAR profile fluctuations of a corner-chamfered meander-line body-implantable antenna operating at 2.5 GHz. For correct dependence analysis, an ANN with two input variables (ɛ_eff_, σ_eff_) and five output variables (SAR_1 g, SAR_10 g, fractional bandwidth, CLM, and DLM) is trained on 80% of all situations and tested on 20% of them. The greatest changes in 1 g and 10 g SAR value, fractional bandwidth, CLM, and DLM at a 4 m distance for 100 Kbps were 63%, 41.6%, 17.97%, 26.79%, and 5.89%, respectively, when compared to the reference effective electrical properties of the homogeneous human body model. The modified antenna architecture results in a change in the antenna’s gain. However, the antenna gain (G_Tx_) in Equation (4) will change if the implantation depth and location change. Additionally, there will be variations in the operating frequency and, consequently, the wavelength (λ). Consequently, the link margin will be computed by entering the acquired G_Tx_ and λ into Equation (4). The range of fluctuation with respect to the mean SAR value is nearly the same in every situation because SAR is reliant on tissue conductivity and density; however, the peak SAR values will change due to changes in antenna construction and implant position. With respect to simulation and measurement, the designed ANN can predict the communication performance of implanted antennas with a respective average accuracy of 99.89% and 99.78% for a possible variation of ±20% in both electrical attributes. For 1 g and 10 g SAR profiles, the respective average accuracies of implantable antennas are 99.01% and 98.27%.

## Figures and Tables

**Table 1:** Comparison with previously reported works.

Ref.	Freq. (GHz)	S_11_ (dB)	Gain (dBi)	SAR_1 g (1 W) (W/kg)	CLM (dB)	DLM (dB)	Comm. Range (m)	Uncertain Parameters	Samples	Variation Analysis Techniques	Variation in Parameters Tested
[32]	2.4	−25	−24.7	697.5	No	No	17 (7 kbps), 4.3 (100 kbps) and 1.4 (1 Mbps)	No	No	No	No
[33]	0.4	−20	−18.9	No	No	No	No	No	No	No	No
[34]	0.915, 2.45	−25, −40	−30.47, −24.71	658, 589	No	No	2	No	No	No	No
[35]	0.915, 2.45	−20, −37	−36, −30.1	333	No	No	No	No	No	No	No
[37]	0.915	−20	−23.23	270.3	No	No	13	Effective permittivity	6	Cartesian plot	Sensing Performance
[38]	0.915, 2.45	−19, −15	−26.30, −20.9	306.19, 252.36	No	No	8	No	No	No	No
[39]	0.402, 2.45	No	−37, −24.5	No	No	No	No	Phantom Size	2	Cartesian plot	Gain, efficiency
[36]	2.45	−11	No	No	No	No	No	Relative Permittivity	6	Cartesian plot	S_11_ and frequency
This Work	2.5	−45.9	−38.42	220.26	20.73 (d = 1 m, Ts = 13 K) (First)	9.28 (d = 1 m, Ts = 13 K) (First)	15 (7 kbps), 10 (100 kbps) and 3.5 (1 Mbps)	Effective permittivity and Conductivity	2500	ANN modeling (First)	CLM, DLM, bandwidth and SAR performance (First)

**Table 2:** Parameters of implantable antenna.

Parameters	Values	Parameters	Values
*l*	20 mm	*w*2	4.397 mm
*w*	10 mm	*w*3	1.46 mm
*l*1	8 mm	*w*4	0.86 mm
*w*1	3.6 mm	*φ*	45°
*l*2	7.4 mm	*P*	(7.25 mm, 2 mm)

**Table 3:** Dielectric properties of different human tissues at 2.5 GHz [42].

Layer	Relative Permittivity	Conductivity (S/m)
Skin	3.8	1.46
Fat	5.28	0.1
Muscle	54.8	2.26
Cortical Bone	11.4	0.39
Cancellous Bone	36.2	1.21

**Table 4:** Parameters for link budget analysis.

	Parameters	Variable	Values
Transmitter	Frequency	*f_r_*	2.5 GHz
	Transmitted Power	*P*_TX_	8.45 dBm
	Tx Antenna Gain	*G*_TX_	−38.42 dBi
Receiver	Receiving Antenna Gain	*G*_RX_	4.95 dBi
	Polarization	*P*	LP
	Temperature	*T_o_*	293 K
	Boltzmann Constant	*K*	1.38 × 10^−23^
	Noise Power Density	*N*_o_	199.95 dB/Hz
Signal Quality	Distance	*d*	1–15 m
	Ideal-BPSK	*E*_b_/*N*_o_	9.6 dB
	Coding Gain	*G*_C_	0
	Fixing Deterioration	*G*_D_	2.5 dB

**Table 5:** Comparison of estimated responses of ANN with simulated and measured results.

Effective Properties		Simulation			ANN			Measurement			% Error of Prediction					
ɛ_eff_	σ_eff_	BW (%)	CLM (dB)	DLM (dB)	BW (%)	CLM (dB)	DLM (dB)	BW (%)	CLM (dB)	DLM (dB)	BW		CLM		DLM	
											Sim	Meas	Sim	Meas	Sim	Meas
44.13	2.10	6.18	13.86	6.12	6.26	13.84	6.08	5.27	13.48	6.21	0.01	0.16	0.08	4.47	0.66	2.14
46.02	0.92	5.58	26.06	16.98	5.62	26.02	17.57	4.96	25.97	17.24	0.01	0.12	0.14	0.27	3.36	1.88
46.02	3.56	7.07	0.71	−4.22	7.13	0.67	−4.25	6.94	0.24	−4.38	0.01	0.03	0.14	5.22	0.71	3.06
49.80	1.80	6.79	17.11	8.94	6.85	17.02	8.97	6.48	16.85	8.85	0.01	0.05	1.52	2.12	1.79	3.36
51.69	1.51	6.88	19.06	9.58	6.91	18.99	9.77	6.72	18.78	9.48	0.00	0.03	0.64	1.92	1.94	2.97
53.59	1.80	7.37	15.98	8.66	7.43	15.95	8.64	7.24	15.76	8.12	0.01	0.03	0.11	2.48	0.23	6.02
55.48	2.10	7.37	13.46	6.54	7.43	13.44	6.47	7.19	13.37	6.78	0.01	0.03	0.08	0.64	1.08	4.79
57.37	0.63	7.88	28.36	20.75	7.75	28.25	20.65	7.52	28.12	20.45	0.02	0.03	1.67	1.73	0.48	0.97
59.26	0.92	7.81	23.11	17.02	7.69	22.95	16.89	7.72	23.04	16.95	0.02	0.00	2.02	1.52	0.77	0.36
63.04	2.97	6.72	4.26	0.65	6.89	4.21	0.67	6.65	3.97	0.64	0.02	0.03	0.27	3.34	2.99	4.48

**Table 6:** Comparison of SAR profile predicted by ANN with simulated results.

Sample	Effective Properties		Simulation		ANN		% Error of Prediction w.r.t Simulation	
	ɛ_eff_	σ_eff_	1 g	10 g	1 g	10 g	1 g	10 g
1	46.02	0.63	250.15	65.09	247.60	64.98	1.03	0.17
2	46.02	0.92	147.78	43.57	145.98	42.65	1.23	2.16
3	46.02	3.56	302.63	74.26	300.98	75.95	0.55	2.23
4	49.80	1.80	227.38	61.01	227.22	61.42	0.07	0.67
5	51.69	1.51	203.46	56.43	202.65	57.23	0.40	1.40
6	53.59	1.80	225.20	60.72	226.18	60.55	0.43	0.28
7	55.48	2.10	348.30	64.03	345.45	65.22	0.83	1.82
8	57.37	0.63	109.15	33.91	108.45	33.22	0.65	2.08
9	59.26	0.92	127.85	42.47	125.65	42.58	1.75	0.26
10	63.04	2.97	231.16	68.81	235.22	69.18	1.73	0.53

## Data Availability

The original contributions presented in this study are included in the article. Further inquiries can be directed to the corresponding author.
